# Knowledge landscape of Treg research in breast cancer: a bibliometric and visual analysis

**DOI:** 10.3389/fonc.2024.1448714

**Published:** 2024-11-27

**Authors:** Zankai Wu, Yanting Zhang, Yiping Gong, Jin Hu

**Affiliations:** ^1^ Department of Breast and Thyroid Surgery, Renmin Hospital of Wuhan University, Wuhan, China; ^2^ Department of Ultrasound Medicine, Union Hospital, Tongji Medical College, Huazhong University of Science and Technology, Wuhan, China

**Keywords:** bibliometrics, Treg, breast cancer, CiteSpace, VOSviewers

## Abstract

**Background:**

Regulatory T (Treg) cells play a strategic role in maintaining immune homeostasis and their functions are closely linked to the development of different diseases, including cancer. This study aims to investigate the evolution patterns and popular research topics of Treg cells through bibliometric analysis.

**Method:**

The Web of Science Core Collection database was used to extract publications related to Treg cells, which were then subjected to bibliometric analysis and visualization through VOSviewer, CiteSpace, and R software.

**Results:**

Between 2003 and 2023, a total of 666 articles were published. China and the United States had the highest citation counts, with Fudan University, Shanghai Jiao Tong University, and Tarbiat Modares University being the leading research institutions. Beckhove Philipp from the German Cancer Research Center and the National Center for Tumor Diseases in Heidelberg, and Christophe from the Cancer Research Center of Lyon, were the most prolific authors. Sakaguchi Shimon from the Immunology Frontier Research Center at Osaka University was the most cited author. “Frontiers in Immunology” published the most articles, while “Journal of Immunology” received the highest co-citations. Key terms in Treg research include immunotherapy, tumor microenvironment, prognosis, immunosuppression, and PD-L1. Among these, immunotherapy, prognosis, PD-L1, and immunosuppression have emerged as focal points of research in recent years.

**Conclusion:**

With active collaboration worldwide, research on Treg cells is rapidly advancing. Focusing on Treg cells as a potential target for cancer treatment shows great promise for future research, especially in terms of practical applications. This could offer valuable direction and fresh perspectives for further exploration of Treg cells in the medical field.

## Introduction

Breast cancer (BC) is the most common cancer in women, with over 1 million new cases diagnosed worldwide each year (1). The incidence of BC has steadily increased in developed countries over the past few decades. BC exhibits certain unique characteristics in terms of age-related incidence rates (2), encompassing a spectrum of diseases with significant differences in morphology, presentation, molecular characteristics, clinical behavior (3), biological features, and treatment response (4). In recent years, the mortality rate attributed to BC has decreased. This is partly due to improvements in screening techniques, surgical and radiation interventions, understanding of disease mechanisms, and more effective use of traditional chemotherapy (5). On the other hand, clinical and pathological factors serve as reliable indicators for treatment decisions and predicting outcomes. These factors encompass tumor size, lymph node involvement, histological grade, vascular invasion, tumor type, as well as patient age and menopausal status (3).

Over the past few years, there has been a growing body of evidence indicating that regulatory T cells (Tregs), a subset of T cells, are vital for upholding tolerance to self-antigens. Tregs were first described in 1995 (6), where they were reported to be involved in the regulation of immune responses and cell activation. Treg cells encompass populations with different phenotypes, cytokine secretion profiles, and mechanisms of suppression (7–9). Several subpopulations of Treg cells, including CD4^+^ Treg cells, CD8^+^ Treg cells, and γδ-TCR Treg cells, were identified and characterized. According to published literature, Tregs are present in cancer and other diseases (10). Additionally, some researchers suggest that Tregs may also be generated by tumor-derived T cells (11, 12). These studies indicate that Tregs are implicated in the tumorigenesis and progression of primary cancers, potentially leading to poor prognosis in cancer patients with BC. Compared to adjacent normal tissues, the mRNA expression of FoxP3, TGFβ1, CCL22, and IL-10 is significantly elevated in cancer tissues. For instance, in progesterone receptor (PR)-negative or HER2-positive tumors, the mRNA expression of FoxP3 and IL-10 is significantly upregulated (13). Furthermore, breast cancer patients exhibit an increase in the number of Tregs in their peripheral blood (14–16), and these regulatory cells are also identified in primary tumors (14).

Bibliometrics, an interdisciplinary science that utilizes mathematics and statistics, offers a thorough and unbiased evaluation of knowledge sources (17–19). Through bibliographic analysis, researchers can gain insights into the progression of a particular topic and uncover trends within the field. This study aimed to explore the landscape of Treg in order to offer fresh perspectives and potential directions for future research in the field.

## Methods

### Search strategies

**Figure 1:**
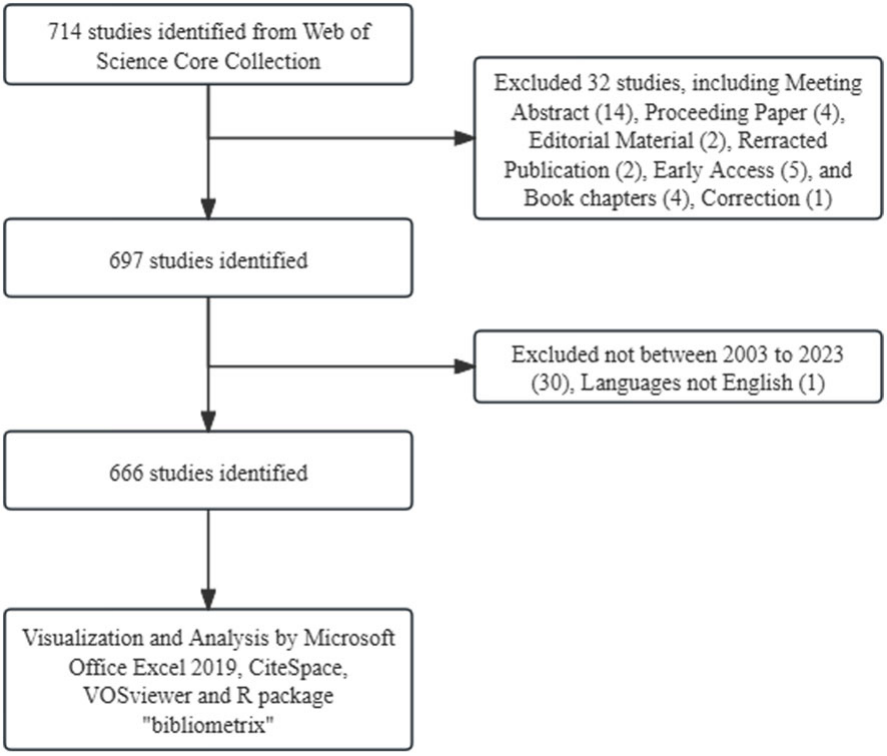
Publications screening flowchart from the Web of Science Core Collection database.

The Web of Science Core Collection (WoSCC) database is extensively employed for academic and bibliometric analysis. Using the query TS=(“Treg”) OR (“regulatory T cells”), we extracted English original articles or review articles published between 2003 and 2023 (**Figure 1**). In order to guarantee the accuracy of the findings, two researchers carried out the literature search and data collection independently.

### Data collection

From the selected publications, we extracted the original research data, which included the journal, publication year, title, authors, country/region, affiliations, abstract, keywords, and references. From the Web of Science, we gathered the journal impact factor (IF) and Journal Citation Reports (JCR) category. Productivity was measured by the number of citations. After exporting the data, we first cleaned it by merging duplicate items into a single entry and manually correcting misspelled words. The data was then subjected to further analysis.

### Bibliometric analysis

R software was employed to carry out Lotka’s Law analysis (20), a technique utilized for quantitatively describing and evaluating the characteristics of literature and its trends using bibliometric indicators. A bibliometric tool like VOSviewer can be utilized to generate scientometric network and knowledge visualizations (21). VOSviewer’s network graph showcases nodes sized by publication count, clustering closely related nodes together. Connections between nodes signify associations, with the strength of the association determining the thickness of the connection. Centrality is a metric used to assess the significance of a node’s position in the network, with nodes having centrality above 0.1 typically viewed as crucial. The CiteSpace software offers a fresh perspective on identifying research hotspots in the Treg field (22).

## Results

### Quantitative analysis of publication

**Figure 2:**
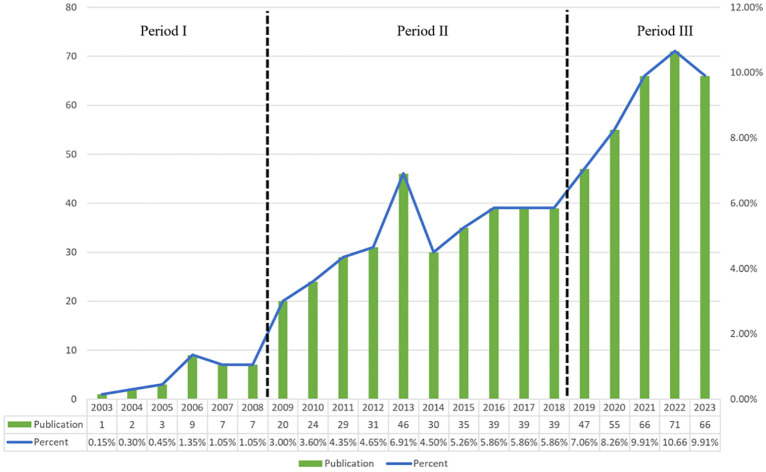
Annual output of research of Treg in BC. The timeframe was divided into three segments: Period I (2003-2008), Period II (2009-2018), and Phase III (2019-2023).

Our search strategy identified 666 studies on Treg in BC in the past twenty years, with 583 being “articles” and 83 “reviews”. By examining the annual growth rate of those publications, we can divide the timeframe into three segments: Period I (2003–2008), Period II (2009-2018), and Phase III (2019-2023). As shown in **Figure 2**, there is a limited number of publications in Phase I, with an average of approximately 4.8 publications per year. This suggests that research on Treg in BC is just beginning. The average number of publications per year was higher in Phase II than in period I, reaching approximately 33.2. As for Phase III, there was a notable increase in publications, averaging about 61 per year. In 2022, there were 71 relevant publications, which was 1.51 times the number published in 2019. In the last five years (Phase III), there has been a steady rise in the quantity of publications about Treg in BC, showing a notable growth in overall papers when compared to the earlier two phases. These findings indicated that Tregs has gained great interest and entered the phase of rapid development.

### Country and institutional analysis

**Figure 3:**
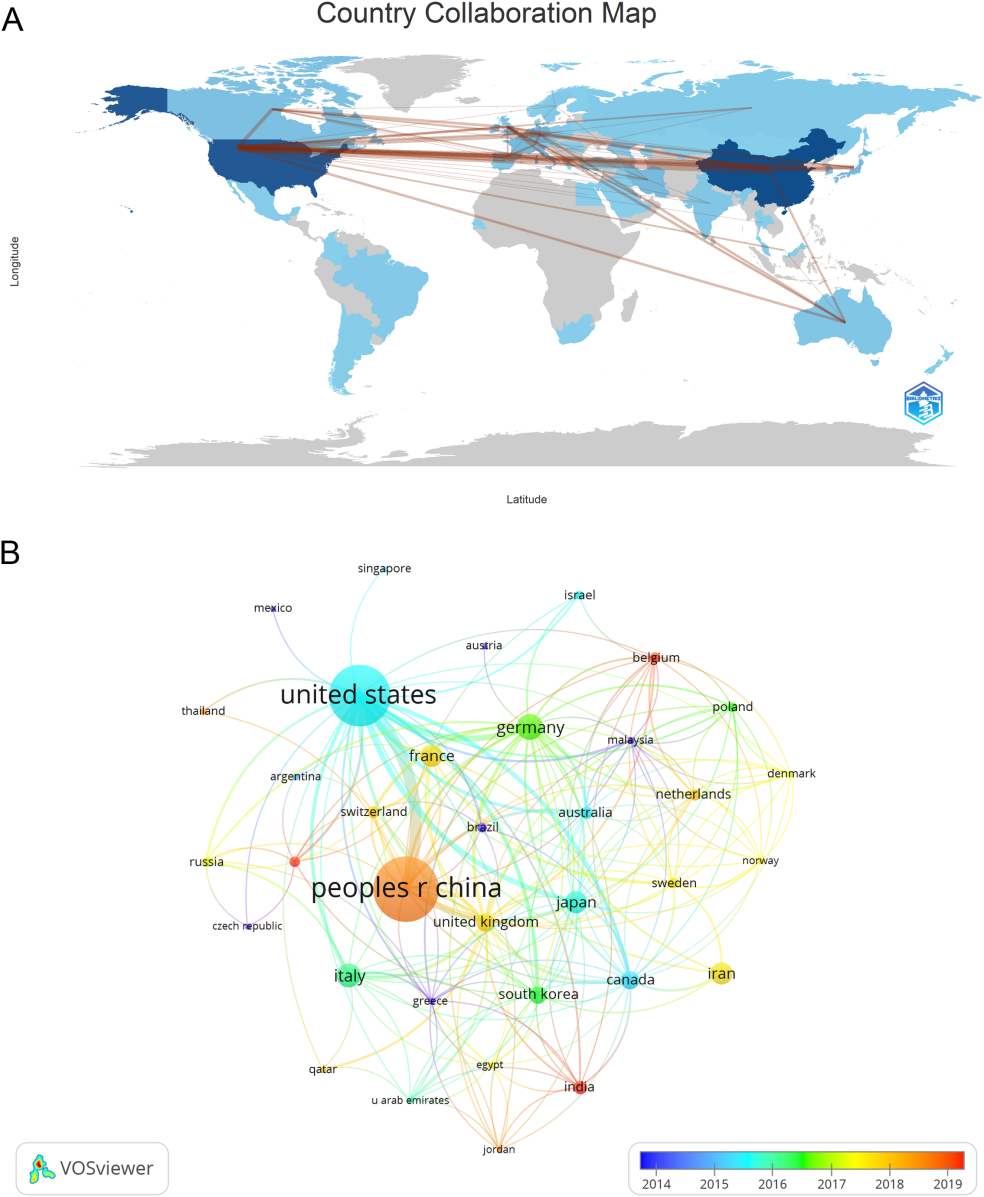
The geographical distribution **(A)** and visualization of countries **(B)** on research of Treg in BC.

The publications analyzed in this study were sourced from 57 different countries and 1119 institutions. The top ten countries represented in the data come from Asia, North America, and Europe, with a significant presence in Asia (n = 322) and America (n = 230) (**Table 1**). China stands out as the country with the most publications (n = 229, 34.38%), followed by the United States (n = 206, 30.39%), Germany (n = 47, 7.05%), and Italy (n = 39, 5.85%). The total number of publications from China and the United States together made up nearly two-thirds of the overall amount (65.31%). Following this, we analyzed and presented data from 57 countries with 2 or more publications, creating a collaborative network based on the quantity and connections of publications in each country (**Figure 3**). A significant point to mention is the extensive cooperation among different countries. For instance, China has strong partnerships with the United States, Canada, and Japan, and the United States actively collaborates with Italy, Germany, Japan, and Iran, indicating that these countries play a crucial role in the research in this field.

**Table 1:** Top 10 countries and institutions on research of Treg in BC.

Rank	Country	Counts	Institution	Counts
1	China (Asia)	229 (34.38%)	Fudan University (China)	16 (2.49%)
2	United states (North America)	206 (30.93%)	Shanghai Jiaotong University (China)	15 (2.25%)
3	Germany (Europe)	47 (7.05%)	Tarbiat Modares University (Iran)	13 (1.95%)
4	Italy (Europe)	39 (5.85%)	University of Pittsburgh (US)	13 (1.95%)
5	Japan (Asia)	36 (5.40%)	Shiraz University Medicine science (Iran)	12 (1.80%)
6	France (Europe)	35 (5.25%)	Tehran University of Medical Sciences (Iran)	12 (1.80%)
7	Iran (Asia)	34 (5.10%)	Inserm (France)	11 (1.65%)
8	United kingdom (Europe)	26 (3.90%)	Nanjing Medicine University (China)	11 (1.65%)
9	Canada (North America)	24 (3.60%)	Shahid Beheshti University of Medical Sciences (Iran)	11 (1.65%)
10	South korea (Asia)	23 (3.45%)	Soochow University (China)	11 (1.65%)

**Figure 4:**
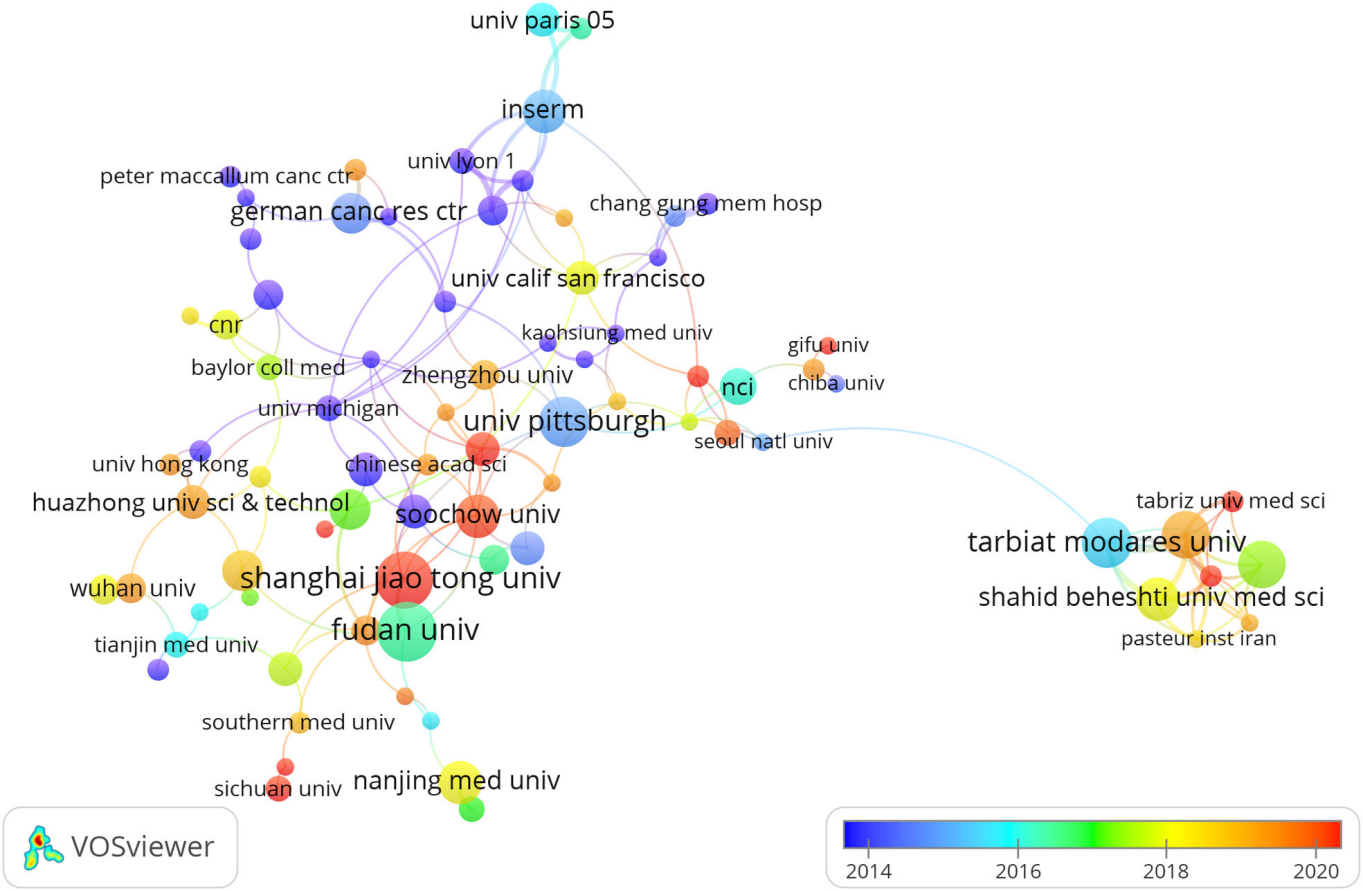
The visualization of institutions on research of Treg in BC, which indicated that these countries play a crucial role in the research in this field.

Four countries are represented among the top 10 institutions, with China and Iran each hosting 40% of them. The leading institutions in terms of relevant paper publications are Fudan University (n = 16, 2.40%), Shanghai Jiaotong University (n = 15, 2.25%), Tarbiat Modares University (n = 13, 1.95%), and University of Pittsburgh (n = 13, 1.95%). Afterwards, we chose 87 institutions that had at least 4 publications for visualization purposes. We then created a collaborative network by analyzing the number and connections between publications from each institution (**Figure 4**). The collaboration among Shanghai Jiaotong University, Fudan University, Tongji University, and Sun Yat Sen University is depicted in **Figure 4**, illustrating their strong partnership. Additionally, there is a robust partnership between Shahid Beheshti University Medical Sciences, Tehran University of Medical Sciences, and Tarbiat Modares University.

### Journals and co-cited journals

**Figure 5:**
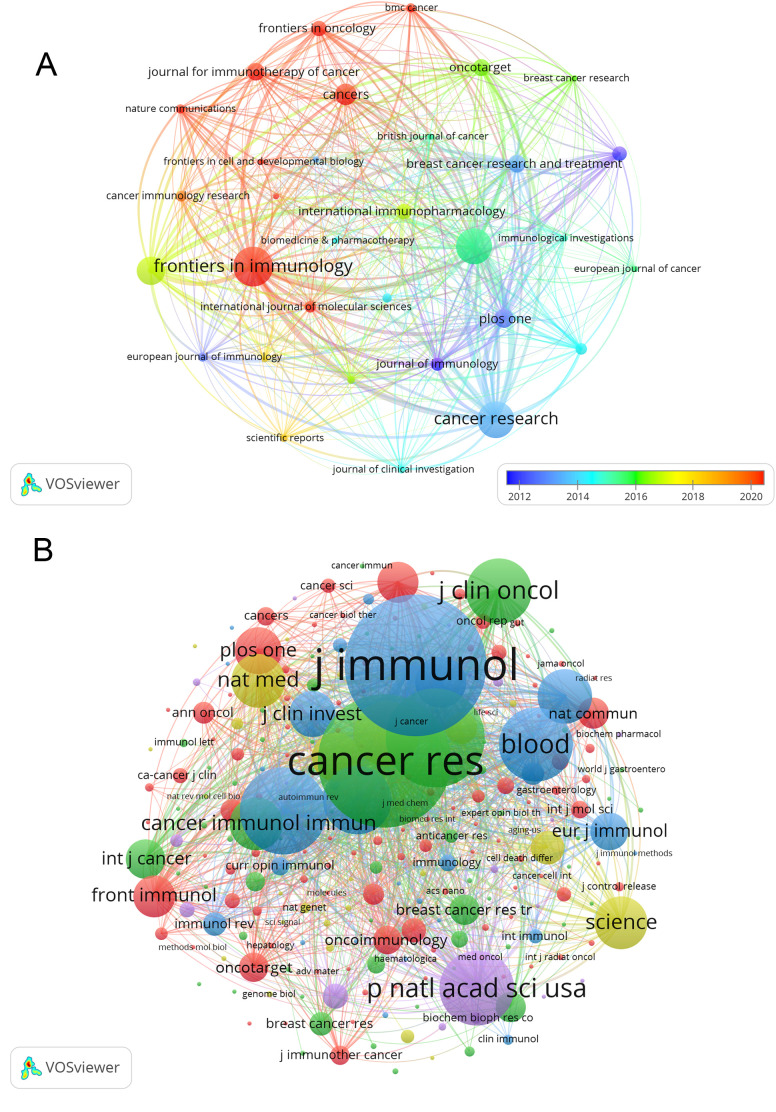
The visualization of journals **(A)**, which illustrates the active citation relationships of Frontiers in Immunology with journals such as Oncoimmunology, Cancer Immunology Research, and Cancers, and co-cited journals **(B)**, which showed that Clinical Cancer Research is positively associated with Cancer Immunology Immunotherapy, Cancer Research, Breast Cancer Research and Treatment, on research of Treg in BC.

Publications on Treg in BC were found in 267 journals. The journal with the highest number of papers was Frontiers in Immunology, with 33 papers (4.9%), followed by Cancer Research with 30 papers (4.5%), Cancer Immunology Immunotherapy with 29 papers (4.4%), and Oncoimmunology with 22 papers (3.3%). The highest impact factor among the top 15 journals is found in Clinical Cancer Research (IF=13.801), followed closely by Cancer Research (IF=13.312). Afterward, we analyzed 32 journals with a minimum of 4 relevant publications and visualized the journal network in **Figure 5A**. This figure illustrates the active citation relationships of Frontiers in Immunology with journals such as Oncoimmunology, Cancer Immunology Research, and Cancers.

According to **Table 2**, out of the top 15 co-cited journals, 4 had more than 1000 citations each. The Journal of Immunology had the highest number of citations (Co-citation=1836), followed by Cancer Research (Co-citation=1707), Clinical Cancer Research (Co-citation=1197), and Journal of Experimental Medicine (Co-citation=1036). Additionally, Nature Reviews Immunology holds the top spot for impact factor at 108.555, while Nature Medicine comes in second at 87.241. Journals with at least 20 co-citations were included in the co-citation network analysis (**Figure 5B**). The co-citation network illustrated in **Figure 5B** demonstrates that Clinical Cancer Research is positively associated with Cancer Immunology Immunotherapy, Cancer Research, Breast Cancer Research and Treatment, and more.

**Table 2:** Top 15 journals and co-cited journals for research of Treg in BC.

Rank	Journal	Count	IF (2022)	Q	Co-cited Journal	Co-citation	IF (2022)	Q
1	Frontiers in Immunology	33	8.786	Q2	Journal of Immunology	1836	5.426	Q2
2	Cancer Research	30	13.312	Q1	Cancer Research	1707	13.312	Q1
3	Cancer Immunology Immunotherapy	29	6.630	Q2	Clinical Cancer Research	1197	13.801	Q1
4	Oncoimmunology	22	7.723	Q2	Journal of Experimental Medicine	1036	17.579	Q1
5	Cancers	16	6.575	Q2	Proceedings of the National Academy of Sciences of the United States of America	855	12.779	Q1
6	Plos One	14	3.752	Q3	Blood	849	25.476	Q1
7	Breast Cancer Research and Treatment	13	4.624	Q2	Immunity	778	43.474	Q1
8	Journal for Immunotherapy of Cancer	13	12.469	Q2	Nature	734	69.504	Q1
9	Oncotarget	12	4.345	Q3	Journal of Clinical Oncology	732	50.717	Q1
10	Frontiers in Oncology	11	5.738	Q3	Nature Immunology	595	31.250	Q1
11	International Immunopharmacology	11	5.714	Q2	Nature Medicine	577	87.241	Q1
12	Clinical Cancer Research	10	13.801	Q1	Cancer Immunology Immunotherapy	569	6.630	Q2
13	Journal of Immunology	9	5.426	Q2	Science	568	63.714	Q1
14	International Journal of Molecular Sciences	8	6.208	Q2	Nature Reviews Immunology	547	108.555	Q1
15	Journal of Translational Medicine	8	8.440	Q2	Cell	518	66.850	Q1

### Authors and co-cited authors

**Figure 6:**
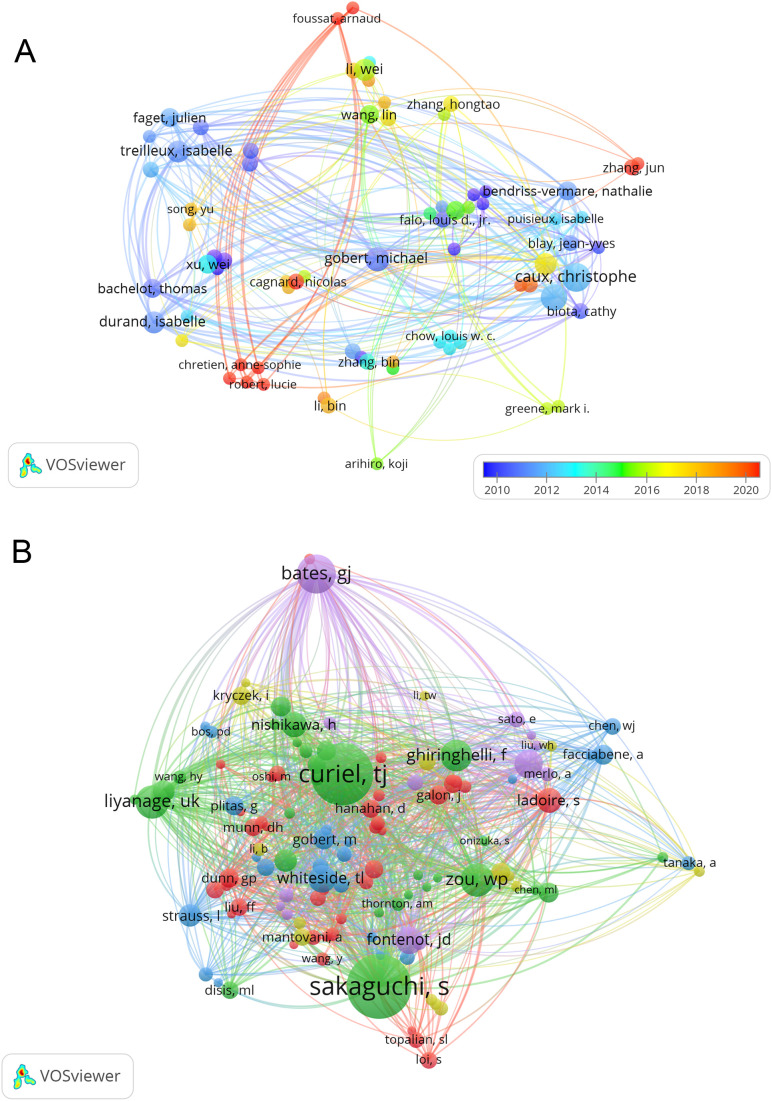
The visualization of authors **(A)** and co-cited Authors **(B)** on research of Treg in BC.

In the study of exosomes in AIDs, a total of 4773 authors took part. The top 10 authors, with five authors each having 6 publications, were highlighted in **Table 3**. To visualize collaboration, we created a network that included authors who had published 2 or more papers (**Figure 6A**). Caux Christophe, Beckhove Philipp, Ghaderi Abbas, Menetrier-Caux Christine, and Sa Gaurisankar stand out with the largest nodes as a result of their prolific publication output. Furthermore, we have observed a significant level of collaboration among multiple authors. For example, Foussat Arnaud collaborates closely with Chretien Anne-Sophie and Robert Lucie, and Wei Li actively partners with Bin Zhang, Yu Song, and Zheng Wang.

**Table 3:** Top 10 authors and co-cited authors of on research of Treg in BC.

Rank	Author	Count	Co-cited author	Citations
1	Beckhove, philipp	8	Sakaguchi, Shimon	203
2	Caux, christophe	8	Curiel, TJ	194
3	Ghaderi, abbas	7	Bates, GJ	111
4	Menetrier-caux, christine	7	Zou, wp	94
5	Sa, gaurisankar	7	Liyanage, uk	93
6	Domschke, christoph	6	Ghiringhelli, f	88
7	Gobert, michael	6	Hori, s	77
8	Peng, guangyong	6	Whiteside, tl	75
9	Schuetz, florian	6	Fontenot, jd	74
10	Durand, isabelle	5	Ladoire, s	65

Out of the 19671 co-cited authors, 5 of them stood out with more than 90 co-citations each. The most referenced author is Sakaguchi Shimon (n = 203), followed by Curiel, TJ (n = 194) and Bates GJ (n = 111). To create co-citation network graphs, only authors with a minimum of 20 co-citations were considered. The resulting **Figure 6B** illustrates the collaborative efforts among different co-cited authors, including partnerships like Sakaguchi Shimon and Curiel TJ, Ghiringhelli F, and Liyanage UK.

### Co-cited references

**Figure 7:**
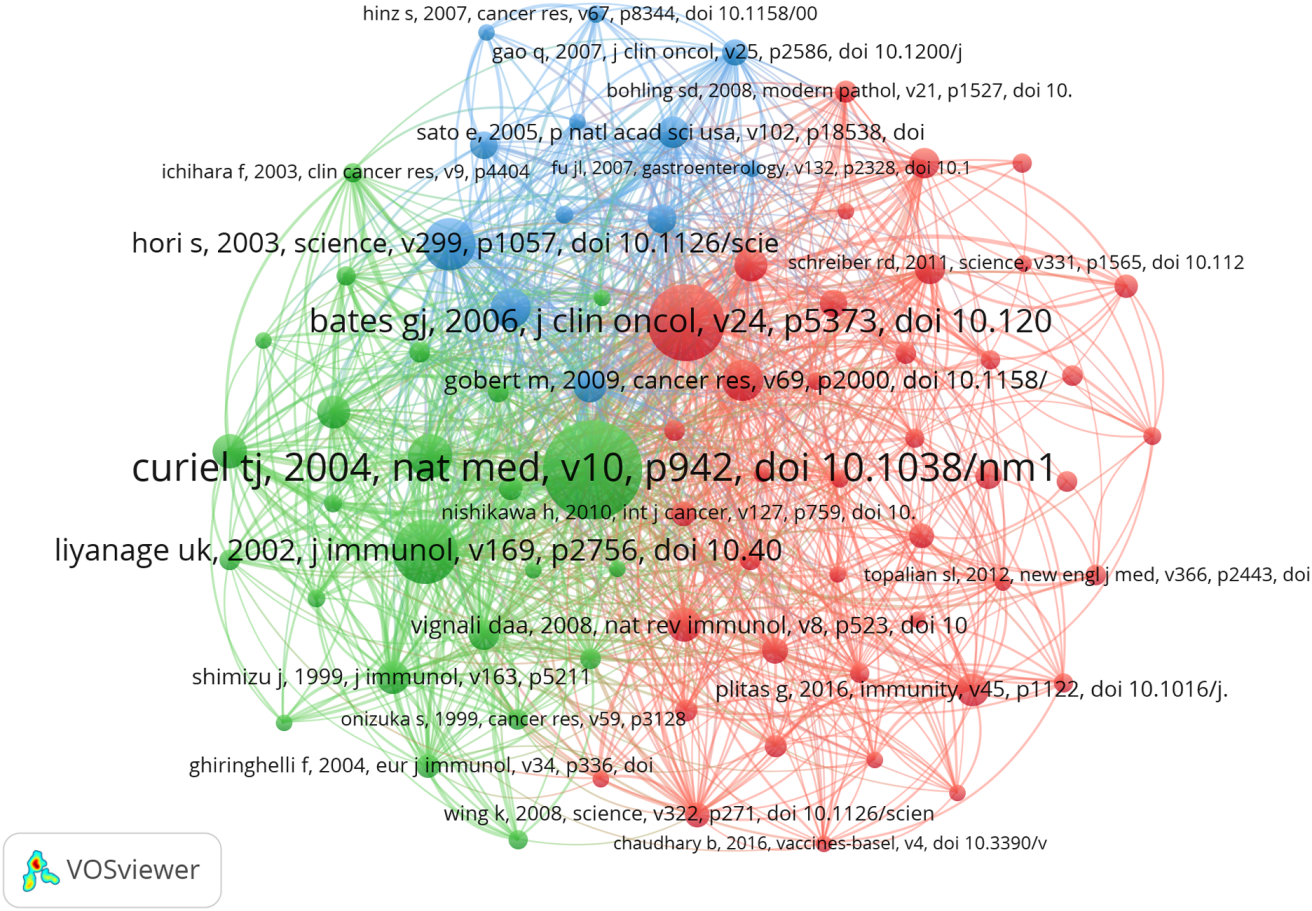
The visualization of co-cited references on research of Treg in BC.

In the research of Treg in BC over the past twenty years, there have been a total of 26330 co-cited references. In **Table 4**, the top 10 co-cited references have all been cited a minimum of 41 times, with one reference having more than 140 co-citations (23). For the construction of the co-citation network map (**Figure 7**), we have chosen references with a co-citation count of 18 or higher. **Figure 7** illustrates that “Curiel TJ, 2004, Nat Med, v10” has active co-cited relationships with references like “Liyanage UK, 2002, J Immunol”, “Zou WP, 2006, Nat Rev Immunol”, and “Ghiringhelli F, 2007, Cancer Immunol Immun, v56”.

**Table 4:** Top 10 co-cited references on research of Th2 in BC.

Rank	Co-cited reference	Citations
1	Curiel tj, 2004, nat med, v10, p942, doi 10.1038/nm1093	147
2	Bates gj, 2006, j clin oncol, v24, p5373, doi 10.1200/jco.2006.05.9584	110
3	Liyanage uk, 2002, j immunol, v169, p2756, doi 10.4049/jimmunol.169.5.2756	89
4	Hori s, 2003, science, v299, p1057, doi 10.1126/science.1079490	69
5	Zou wp, 2006, nat rev immunol, v6, p295, doi 10.1038/nri1806	57
6	Gobert m, 2009, cancer res, v69, p2000, doi 10.1158/0008-5472.can-08-2360	53
7	Fontenot jd, 2003, nat immunol, v4, p330, doi 10.1038/ni904	51
8	Vignali daa, 2008, nat rev immunol, v8, p523, doi 10.1038/nri2343	43
9	Woo ey, 2001, cancer res, v61, p4766	42
10	Wolf am, 2003, clin cancer res, v9, p606	41

### Reference with citation bursts

**Figure 8:**
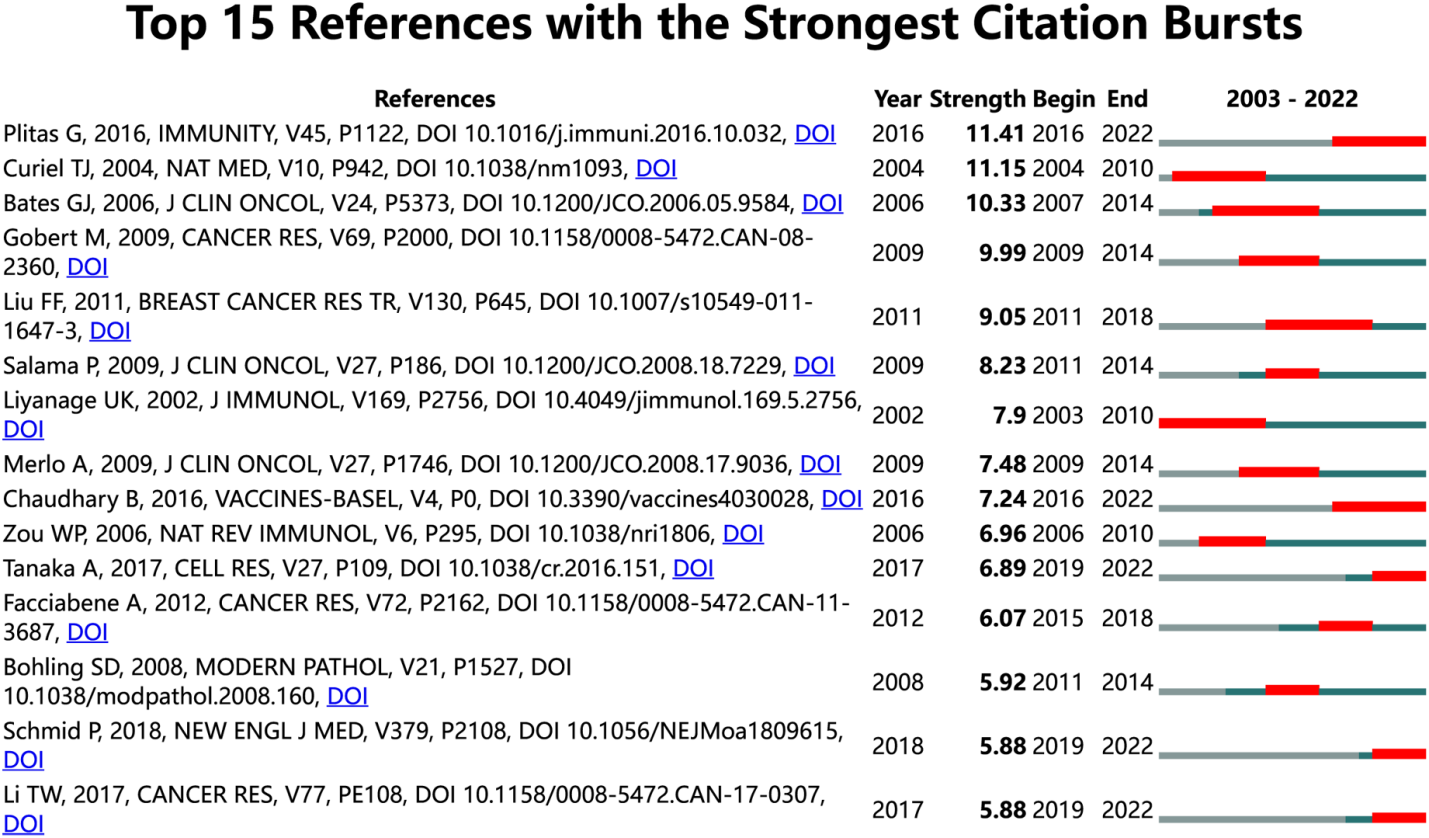
Top 15 references with strong citation bursts. A red bar indicates high citations in that year. In general, the 15 references had burst strengths ranging from 5.88 to 11.41 and endurance strengths between 3 to 7 years.

In our study, we identified 15 references with strong citation bursts, which are frequently cited by scholars in a specific field over time. CiteSpace helped us pinpoint these references, as depicted in **Figure 8**, where the red bar signifies significant citation burstiness (24). From 2013 to 2019, citation bursts for references were noted. The reference “Regulatory T Cells Exhibit Distinct Features in Human Breast Cancer” by Plitas George et al. had the strongest citation burst (strength=11.41), with bursts of citations recorded from 2016 to 2022. Curiel TJ et al. published the reference “Specific recruitment of regulatory T cells in ovarian carcinoma fosters immune privilege and predicts reduced survival” in Nature Medicine. This article had the second strongest citation burst (strength=11.15) from 2004-2010. In general, the 15 references had burst strengths ranging from 5.88 to 11.41 and endurance strengths between 3 to 7 years. **Table 5** outlines the primary research topics covered in these references, following the order of the literature presented in **Figure 8**.

**Table 5:** The main research contents of the 15 references with strong citations bursts.

Rank	Strength	The main research content
1	11.41	Targeting CCR8 for the depletion of tumor-resident Treg cells might represent a promising immunotherapeutic approach for the treatment of breast cancer.
2	11.15	Blocking T-reg cell migration or function may help to defeat human cancer
3	10.33	FOXP3-positive T-R represent a novel marker for identifying late-relapse patients who may benefit from aromatase therapy after standard tamoxifen treatment.
4	9.99	Treg are selectively recruited within lymphoid infiltrates and activated by mature DC likely through TAA presentation, resulting in the prevention of effector T-cell activation, immune escape, and ultimately tumor progression.
5	9.05	The density of intratumoral Treg infiltrates and the peritumoral CTL/Treg ratio are independent prognostic factors and correlated with the prognosis of the molecular subtypes of breast carcinoma
6	8.23	FOXP3(+) Treg density in normal and tumor tissue had stronger prognostic significance in colorectal cancer compared with CD8(+) and CD45RO(+) lymphocytes.
7	7.9	The prevalence of T-reg is increased in the peripheral blood as well as in the tumor microenvironment of patients with invasive breast or pancreas cancers.
8	7.48	FOXP3 expression as a new independent prognostic factor in breast carcinoma, which might help to improve the selection of patients for appropriate therapy.
9	7.24	In this review, they critically examine the role of Tregs in the tumor microenvironment and in cancer progression focusing on human studies.
10	6.96	Strategies for therapeutic targeting of regulatory T cells and the effect of regulatory T cells on current immunotherapeutic and vaccine regimens
11	6.89	FOXP3-expressing regulatory T (Treg) cells, which suppress aberrant immune response against self-antigens, also suppress anti-tumor immune response.
12	6.07	They discussed the ways in which Tregs suppress the antitumor immune response and elaborate on our recent discovery that Tregs make significant direct contributions to tumor angiogenesis.
13	5.92	Regulatory T cells may play a role in inducing immune tolerance to higher grade, more aggressive breast carcinomas, and are a potential therapeutic target for these cancers.
14	5.88	Atezolizumab plus nab-paclitaxel prolonged progression-free survival among patients with metastatic triple-negative breast cancer in both the intention-to-treat population and the PD-L1-positive subgroup.
15	5.88	TIMER provides a user-friendly web interface for dynamic analysis and visualization of these associations, which will be of broad utilities to cancer researchers.

### Hotspots and Frontiers

**Figure 9:**
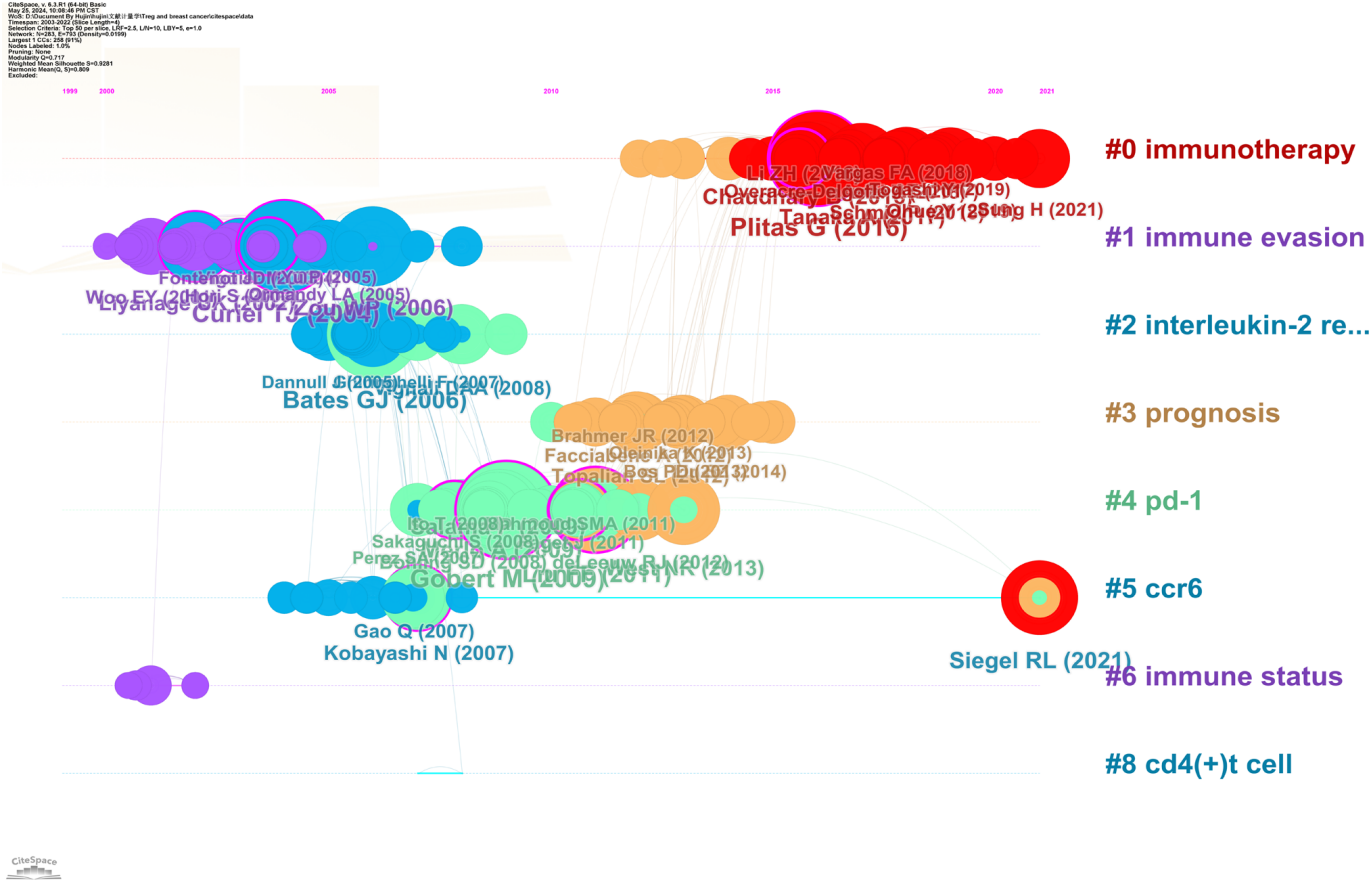
Timeline viewer related to Treg in BC. Eight clusters were identified, covering topics such as immunotherapy, immune evasion, interleukin-2 regulation, prognosis, PD-1, CCR6, immune status, and CD4(+) T cell.

The Timeline viewer allows you to observe the changing trends of research hotspots through keywords, and analyze the temporal patterns of research fields as shown by clusters and the progression of popular keyword research over time. When documents are grouped together in a cluster, they are positioned on a single horizontal line, with time moving from left to right to demonstrate the significance and quantity of research accomplishments within that particular field. Based on the CiteSpace parameters, a network was generated with 283 nodes, 793 connections, and a density of 0.0199. **Figure 9** visually represents the key areas and future directions of research on Treg and BC over time. The Cluster ID indicates the specific clustering number. The clusters are represented as # 0, # 1, # 2, and so on, with the size of each cluster indicating the number of members it contains. In **Figure 9**, there are eight clusters identified, covering topics such as immunotherapy, immune evasion, interleukin-2 regulation, prognosis, PD-1, CCR6, immune status, and CD4(+) T cell.

By analyzing the co-occurrence of keywords, we are able to easily identify the current research trends in a specific field. **Table 6** displays the top 20 frequently used keywords in the study of Treg in BC. Notably, Immunotherapy and Tumor microenvironment were mentioned over 35 times, indicating the primary focus of Treg research in BC.

**Table 6:** Top 20 keywords on research of Treg in BC.

Rank	keywords	Count	Rank	keywords	Count
1	Breast cancer	143	11	T cells	17
2	Regulatory T cells	76	12	PD-L 1	15
3	Immunotherapy	65	13	Tregs	15
4	FOXP3	46	14	Metastasis	14
5	Treg	41	15	Triple-negative breast cancer	14
6	Cancer	39	16	Inflammation	13
7	Tumor microenvironment	38	17	Cancer immunotherapy	12
8	Prognosis	30	18	Colorectal cancer	12
9	Treg cells	29	19	Immunomodulation	12
10	Immunosuppression	18	20	Tumor	12

**Figure 10:**
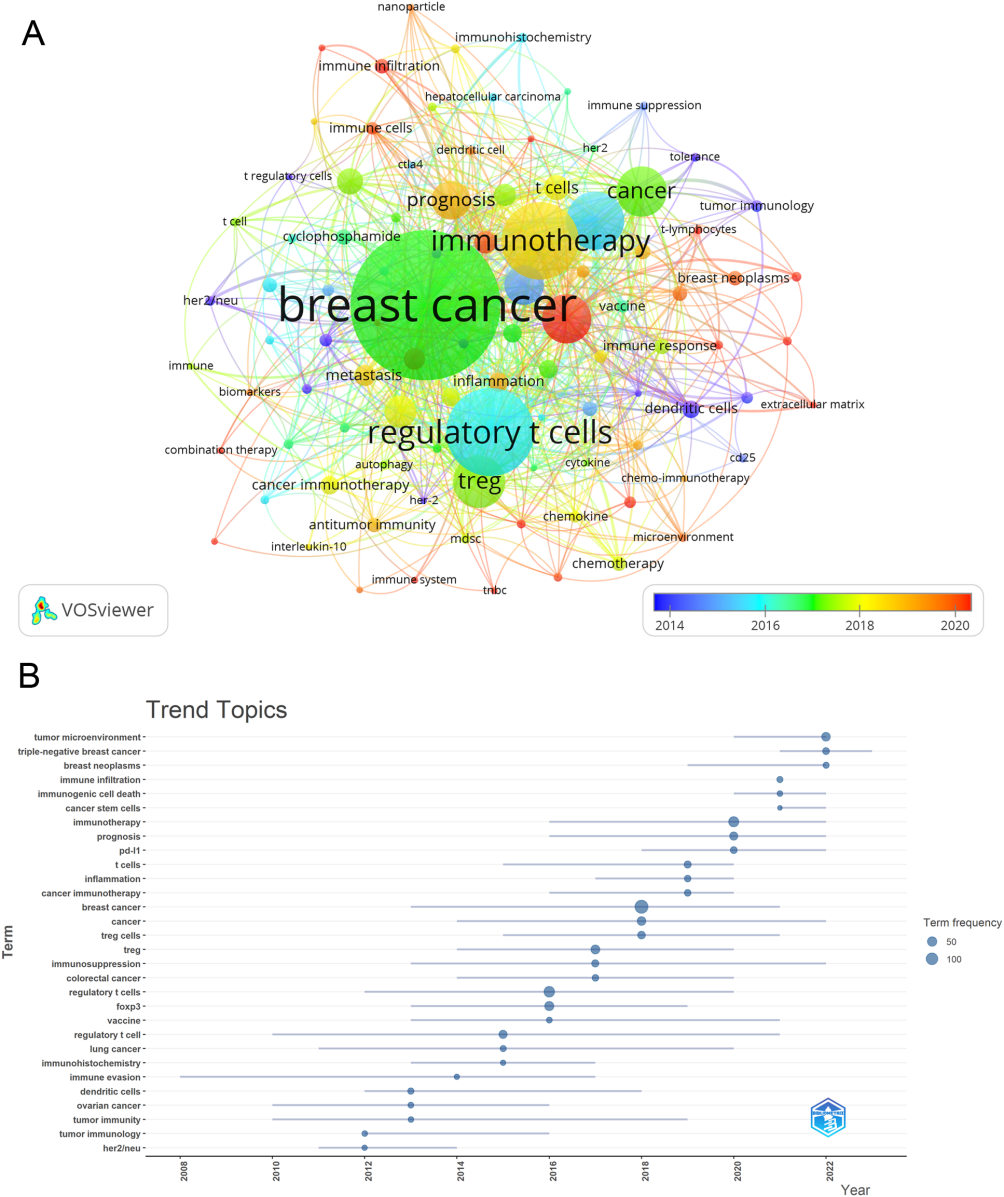
Keyword cluster analysis **(A)**, which showed eight clusters that correspond to eight distinct research directions, and which clusters contain keywords like Immunotherapy, tumor microenvironment, prognosis, Immunosuppression, and PD-L1, and trend topic analysis **(B)**. Main keywords identified during this time were immune evasion, immunohistochemistry, and immunosuppression.

Once we had identified keywords with at least 4 occurrences, we utilized VOSviewer to carry out cluster analysis (**Figure 10A**). The intensity of the relationship between keywords is illustrated by the thickness of the connecting lines. In **Figure 10A**, we found eight clusters that correspond to eight distinct research directions. These clusters contain keywords like Immunotherapy, tumor microenvironment, prognosis, Immunosuppression, and PD-L1. During the period of 2008 to 2022, the analysis of trending topics related to keywords (**Figure 10B**) indicated a primary focus on cancer and tumor immune research. The main keywords identified during this time were immune evasion, immunohistochemistry, and immunosuppression. Scholars have recently delved into the pathogenesis and therapeutic opportunities of Treg in BC, with key areas of interest including PD-L1, inflammation, cancer stem cells, prognosis, immunogenic cell death, and other related factors since 2016. Moreover, over the past three years (2020-2022), the keywords Immunotherapy, tumor microenvironment, prognosis, Immunosuppression, and PD-L1 have been consistently prominent, indicating that they likely reflect the current research focus on Treg in BC.

## Discussion

In BC, Tregs have been shown to have varying prognostic significance among different subtypes, leading to conflicting conclusions in the literature. However, upon comparing different theories, it is more likely that Tregs serve as an unfavorable factor in the overall prognosis of BC (25). Extracted from public databases, this study focused on Treg studies for bibliometric analysis to pinpoint hotspots and development trends, revealing the notable potential of Treg in BC through the rising annual publication volume.

China and the United States had the highest number of citations, but the distribution of institutions did not align with geographical regions. The top 10 institutions were spread across 4 countries (China, Iran, US, and France), with 40% located in China and another 40% in Iran. Despite the average citations not aligning with the number of publications in this field, it is evident that more comprehensive efforts are needed to fully understand the role and mechanism of Treg in BC. It is evident that this scenario will impede the progress of the research field in the future. As a result, we urge research institutions in different countries to collaborate extensively and communicate effectively in order to collectively advance the development of Tregs in BC.

Frontiers in Immunology published the most articles, indicating it is currently the most popular journal in this research field, while Journal of Immunology received the highest number of co-citations. Emerging journals Cancers and Frontiers in Oncology focused on disseminating research on macrophages. Scholars tended to review papers from highly cited journals such as Nature, Blood, and Cell, leading to increased citations for those papers. Obviously, these journals are high-quality international journals, providing support for the study of Tregs in BC.

Beckhove Philipp’s productivity as an author is evident in the eight papers, he published between 2009 and 2021, with a primary focus on Treg clonal origin and the role of antitumor immunity (26–33). On the other hand, Sakaguchi Shimon is the most cited author in the Treg field. Sakaguchi’s main focus was on therapeutic immunity, either through adoptive tumor-primed CD4^+^ T-cells or by examining the ratio of CD8^+^ lymphocytes to tumor-infiltrating cells (34, 35). Alongside his collaborations, Curiel TJ delved into the relationship between sex and cancer via B7-H1 (36). Bates’s research group at the University of Oxford delved deeper into the recruitment of Treg cells in Triple-negative BC (37).

Due to a range of treatments such as hormonal therapy, adjuvant chemotherapy, radiation therapy, and surgical resection, the outlook for patients with BC has significantly improved in recent years. However, the challenges of recurrence, metastasis, and drug resistance persist without clear resolution, and the underlying causes and mechanisms of these occurrences have not been definitively identified. While several risk factors for BC have been studied, this remains an ongoing area of investigation. Treg infiltration is a recognized risk factor for cancer development and death. Individuals with Treg infiltration had worse overall survival, progression-free/disease-free/recurrence-free survival, or breast cancer-specific survival in comparison to those without Tregs. Treg cells are present in high numbers in different types of tumor tissues, including the lung, liver, pancreas, gastrointestinal tract, BC, and malignant melanoma. Their infiltration is linked to a negative prognosis in ovarian, breast, and gastric cancers (38). Tregs found in cancer patients have the ability to identify various tumor antigens, such as survivin and Ny-ESO-1, and are capable of inhibiting tumor antigen-specific T cells (39). The analysis of keyword clustering revealed that research on Tregs spanned from the biological characteristics of macrophages to the focused treatment of cancer. Treg is strongly linked to particular pathological contexts, and its complex role in tumors has been extensively studied. Angiogenesis, which establishes the fundamental conditions for tumor progression, has been a key area of research. New findings indicate that Tregs may have unique roles in influencing both angiogenesis and metastasis in the tumor microenvironment. When exposed to low oxygen levels, Tregs secrete vascular endothelial growth factor A (VEGFA), which stimulates the formation of new blood vessels by promoting the maturation of endothelial cells and initiating angiogenesis (40).

Different subsets of Treg cells, such as CD4^+^ Treg cells, CD8^+^ Treg cells, and γδ-TCR, have been recognized and characterized. Tregs have been observed in cancer and other illnesses (10). CD4^+^ Treg cells derived from tumors have been extensively examined in numerous cancer types. Additionally, the presence of antigen-specific CD4^+^ Tregs within tumors can greatly inhibit immune responses, ultimately resulting in immune tolerance toward tumor cells. While T cells play a crucial role in the early stages of cancer by surveilling and controlling tumor growth, they can transform into suppressive CD4^+^ and CD8^+^ Tregs after prolonged exposure to tumor cells. This transformation ultimately aids in the advancement rather than the inhibition of cancer development and progression (41). In the effector phase of the immune response triggered by tumor-primed CD4^+^ T cells that were transferred adoptively, both tumor Tregs and naive Tregs are unable to suppress antitumor immunity. However, tumor Tregs play a crucial role in suppressing tumor-specific CD8^+^ T cell responses in tumor-draining lymph nodes, thus impeding antitumor immunity at the initial stages of the immune response triggered by adoptively transferred tumor-primed CD4^+^ T cells (42).

The bibliometric study offered a glimpse into the development trend and research hotspots in this field, though it was not without its limitations. The literature analyzed was gathered from the WOSCC database, potentially resulting in the exclusion of pertinent information. Moreover, biases in the bibliometric method utilizing natural language processing could have influenced the results if adjustments were made to compensate for inaccuracies. In addition, the analysis relies solely on the Web of Science, potentially omitting relevant studies from other databases.

In conclusion, the study of Treg is continuously advancing with global collaboration. The development of anticancer therapies targeting Treg is a growing and hopeful area for future research, particularly in translational applications. This could offer valuable guidance and fresh perspectives for further exploration in the field of Treg.

## Data Availability

The original contributions presented in the study are included in the article/supplementary material. Further inquiries can be directed to the corresponding authors.
